# The Physiology and Treatment of Sensitive Dentine

**Published:** 1890-06

**Authors:** George M. Keevil


ARTICLE III.

### THE PHYSIOLOGY AND TREATMENT OF SENSITIVE DENTINE.

BY GEORGE M. KEEVIL.

[A paper read before the Students' Society, National Dental Hospital.]

MR. PRESIDENT AND GENTLEMEN:—Having been asked on several occasions to read a paper before this Society, I have always been obliged to decline on the ground of not being able to find a sufficiently novel subject for it, and although I cannot claim much novelty or originality for the title and matter of to-night's paper, yet it is a subject which is always being brought vividly and practically home to the mind of the dental practitioner, and for that reason I venture to hope that it may be sufficiently interesting to tempt you to sit it out.

In order to treat of this subject, it is necessary to briefly refer to the structure of dentine, well-known though it may be to most of you. This tissue is usually defined as consisting of a hard calcified matrix, permeated by a system of channels, which contain a process or elongation of the odontoblast layer of cells, this layer being in connection with those immediately beneath them, thus establishing an intimate connection between the pulp and dentine. This is alone sufficient to warrant a theory that the sensibility of dentine is dependent upon the pulp, but the fact that Bœdecker has demonstrated the presence of a minute plexus throughout the whole structure of dentine, derived from the ultimate branches of the fibrils, is an additional support to such a theory, dentine thus presenting an appearance of a nervous supply analogous to that of the other tissues of the body.

Salter believed that dentine has a nervous connection both with the pulp and periosteum, and in support of this he mentions instances of dentine retaining an intense sensibility after direct communication with the pulp had been cut off by the lesion of intervening tissue. As he observes, however, it does not necessarily follow that the connection between the pulp and dentine should be by a direct radiation in the course of the dentinal tubes, it may be circuitous, and thus an outlying mass of dentine retain its sentient connection with the pulp; and if we remember Bœdecker's demonstration just mentioned, this latter view would appear very probable, and more so because we can only admit that any connection between the pulp and periosteum could only occur in that portion of the tooth which is enveloped in that membrane. It is interesting, historically, to note that John Hunter did not consider dentine to be a tissue capable of transmitting sensation; he says that "teeth are occasionally worked upon by operators in the living body without pain," but he evidently had not had much experience in the matter. It has never yet been determined how the nerves of the pulp terminate. Tomes states that they terminate in a rich plexus beneath the odontoblast layer. Boll, however, in investigating this point, found that on treating a pulp with dilute chromic acid solution, an immense number of medullated nerve fibres could be traced onward into non-medullated ones, the ultimate destination of which however was uncertain, but he has seen them passing between the odontoblasts and taking a direction parallel to that of the dentinal fibrils in such numbers that he infers that they have been pulled out of the tubes, still, however, he has not seen a nerve fibre definitely to pass into a canal. His observations have been controverted by Magitot, who recently stated that he had fully satisfied himself that the nerves of the pulp become continuous with the stellate layer of cells, and through the medium of these with the odontoblasts. If this latter view be correct, the sensitiveness of dentine would be accounted for, without the necessity that nerves should actually enter it, for the dentinal fibrils would then be prolongations, so to speak, of the nerves, and as Tomes says, it is not necessary to assume that the dentinal fibrils should actually be nerve fibres, for many animals of a low organization are capable of sensation, although they have no demonstrable nervous system.

The foregoing facts plainly point to the conclusion that a certain degree of sensibility in dentine is normal; on histological grounds, there is also the following fact that dentine loses all sensibility on destruction of the pulp. Whether this degree of sensibility varies or not at different ages is uncertain. I have been unable to find statistics bearing on this point or upon another interesting point, and that is, what relation, if any, the sensitiveness of dentine bears to the state of the rest of the nervous system at the time of observation. With regard to the latter question, Dr. White in an article in the *Dental Review* mentions two or three cases in which a highly nervo-sanguineous temperament was accompanied by an exquisitely sensitive state of the dentine, and in one of them in which the children inheriting the parent's temperament were also affected with a like condition; more statistics, however, are required to show whether this was merely a coincidence or not. It is also said that the teeth of young persons, especially those just arriving at the age of puberty, are exceedingly sensitive, and to be more particularly marked in girls. It was formerly supposed that in aged, or senile dentine, the fibrils atrophied or became calcified, and that in consequence sensation was gradually lost, but Wedl proved that they were still there, and also retained their property of imbibition, it is only fair therefore to suppose that they still retained their sensibility. Regarding the uses of the sensations in dentine nothing definite is known. Mr. Coleman is inclined to accord them a special function, probably tactile, by which we are enabled to judge of the nature of substances which come under their action, and also to judge of the requisite amount of mastication, for it is an undoubted fact that persons with artificial teeth find at first considerable difficulty in ascertaining when their food has been sufficiently masticated; but it appears to me that this function, which is entirely dependent upon pressure, is more probably performed by the periosteum. It is suggested that the most likely use of the sensation is to give warning of the encroachings of caries, and to be intimately connected with the throwing out of secondary dentine. Being pretty well agreed, therefore, that a certain degree of sensation in dentine is normal, we must regard any exalted state of such sensibility as a pathological condition, but to what this hyperæsthesia is due is a matter which has been very violently discussed, without any definite conclusions being arrived at, the most usual thing to do in order to get out of the difficulty is to put it down to inflammation. The only thing which can be said against it is that the dentinal tubes are too small to admit blood corpuscles, but even this is a false objection, as there are countless numbers of immature corpuscles, the so-called "microcytes," which are capable of being exuded into the tubes. Thomas Bell, in endeavoring to establish the inflammatory theory says, that in many cases after breaking open a tooth, immediately after extraction, where the pain and inflammation had been severe, he found distinct red patches in the very substance of the dentine. He also states that on the examination of teeth of persons killed by drowning or hanging, he found the whole of the osseous part of the teeth, with the exception of the enamel, tinged a deep red. He places this against the objection of John Hunter and others, that the teeth have never been satisfactorily injected, and he especially mentions the case of a patient who died from jaundice, in which the dentine was found to be of a bright yellow colour. Amongst modern authorities who are in favor of the inflammatory theory may be mentioned Drs. Harris and Taft, but the worst of it is that no treatment based scientifically on this theory produces any satisfactory results.

Another and more plausible suggestion is, that the pulp itself is really the seat of the exalted sensibility, and the fibrils are merely the agents through which external impressions are conveyed to this central organ. Rational treatment based on this hypothesis would be the administration of such drugs, as acting on the nervous or circulatory systems, or both, should lower this exalted. Experiments involving the use of nervous and arterial sedatives have not tended to confirm this theory, although morphia is said to be of use in such cases as those which I have before mentioned in which great sensitiveness of dentine was a peculiarity of a nervous temperament, but in these cases, however, the hyperæsthesis cannot be considered to be a pathological state.

Another suggestion to account for this peculiar condition is, that it is caused by the crowding into the dentinal tubes, of bacteria, when disturbed by an exciting cause, and so causing pressure on the fibrils. Dr. Willmot's opinion is, that hypersensitive dentine as a pathological condition is analogous to that condition known as "teeth on edge," and is produced by the same general cause, the irritation of an acid. He says, that in these cases of "teeth on edge" from eating sour fruit, &c., the acid is concentrated and abundant, passing through pores and cracks in the enamel, acts on the peripheral extremities of the fibrils, and causes such irritability in the dentine that the slightest impact or change of temperature causes pain. He goes on to say that in the hyperæsthesia generally observed in general practice, associated with caries, the irritating acid is very dilute, so that the effect is produced slowly, requiring for its manifestation greater changes of temperature or some chemical or mechanical injury, as the cut of an excavator, the difference of the two conditions being in degree only; in the former the irritant being in action for a short time only, and so soon becoming diluted with saliva, that an exalted sensibility rapidly subsides; in the latter the irritation due to an acid condition of saliva, is persistent, and the hyperæsthesia soon becomes chronic.

I have now to pass to a more practical point, namely, treatment. The internal use of morphia has already been alluded to, the conclusion being that it is only of use in cases of systemic nervousness, and being a dangerous drug to play with should be excluded as much as possible from use by the dental practitioner, the risks not being counterbalanced by satisfactory results. In cases in which the sensibility is referable to an acid state of the secretions, a removal of the cause will at once suggest itself, cases such as these, and which occur so frequently in children, are easily corrected by the use of alkaline remedies, such as bicarbonate of soda or potash.

In such cases as are not included in the above, a lot may be gaining the confidence of the patient, by tact of manner and touch, and by proper use of sharp instruments, but it is in those cases, such as erosion cavities, in which the dentine is so hard as to require the determined use of the dental engine, which gives so much trouble, and to find a remedy for this class of case the whole *Materia Medica* has been ransacked from one end to the other, with very indifferent success. Dehydration by means of absolute alcohol, followed by warm air is one of the first things which should be tried, its apparent action being the subtraction of water from the organic material in the dentinal tubes, causing its contraction.

The next most primitive method of treatment is the application of creasote or carbolic acid, the latter by preferance. Carbolic acid is a drug in which the relative dryness of the cavity and maintenance of the strength of the application produce proportionately satisfactory results, but it requires to remain for two or three hours to do any good. Dr. Flagg considers this drug to act in a two-fold way, both by forming insoluble phenates with the organic constituents of the dentine, and by anæsthetizing to a slight degree the pulp, rendering that organ less susceptible to irritation.

The next on the list of this class of remedies is a very favorite one, chloride of zinc; in using this drug it is absolutely essential that the cavity be as dry as possible, as success depends upon its being diluted, and in order to secure this, it has been recommended to place small pieces of it in the cavity and allow them to deliquesce of their own accord; fifteen minutes is usually sufficient time for this drug to produce its effects, unfortunately, however, its application is usually attented with intense pain, and I am not aware that any means have yet been found for avoiding this. Occasionally, in deep seated cavities, the application of chloride of zinc produces pain of a beating kind; it is an indication that the pulp is being affected, and this should be at once controlled by washing the cavity out with warm water and introducing a little oil of cloves.

Side by side with chloride of zinc may be placed nitrate of silver, which is said to affect the dentine to a greater depth and to be less painful, but the unfortunate discoloration which it produces is an effectual bar to its use, at any rate in front teeth.

The last of this series of drugs is arsenic; but although this substance is undoubtedly the best obtunder at present known, its use is almost unanimously condemned on account of its deleterious effect upon the pulp. If, however, its use is resorted to as a last resource, Dr. Harris recommends 1-40th grain to be applied and allowed to remain for about an hour. It is doubtful, though, whether its employment is justifiable for the purpose of simply obtunding dentine.

Among other remedies may be mentioned tannin in alcoholic solution, in creasote or in glycerine, sulphuric acid and cocaine, oxide of calcium, and the frequent administration of small quantities of nitrous oxide gas, has been said to be efficacious in some cases. These have all been used with more or less success, but their action appears to be very uncertain.

With regard to electricity as applied for the purpose of treating sensitive dentine, Dr. Harris mentions the galvanic cautery as being useful.

Dr. Flagg states that in many cases he has found galvanic electricity of great use in obtunding, but that it does not always bring about the result. He uses it by placing one pole of a large bichromate battery on the gum over the root of the affected tooth, the other being placed in the cavity, each pole for better connection being tipped with sponge; it probably acts by producing a retraction of the organic material in the dentinal tubes, in the same way that electricity is known to cause the contraction of protoplasm, when applied to it as a stimulant.

In concluding my short paper, gentlemen, I trust that you will attribute its brevity to lack of material, not to want of painstaking in trying to get it together.—*London Dental Record.*

